# Non-invasive surveillance of *Plasmodium* infection by real-time PCR analysis of ethanol preserved faeces from Ugandan school children with intestinal schistosomiasis

**DOI:** 10.1186/s12936-019-2748-4

**Published:** 2019-04-01

**Authors:** Hajri Al-Shehri, B. Joanne Power, John Archer, Alice Cousins, Aaron Atuhaire, Moses Adriko, Moses Arinaitwe, Abdullah D. Alanazi, E. James LaCourse, Narcis B. Kabatereine, J. Russell Stothard

**Affiliations:** 1 Department of Tropical Disease Biology, Liverpool School of Tropical Medicine, Liverpool, L3 5QA UK; 2 Ministry of Health, Asir District, Abha, Kingdom of Saudi Arabia; 3 Wellcome Centre for Integrative Parasitology, University of Glasgow, Sir Graeme Davies Building, 120 University Place, Glasgow, G12 8TA UK; 4 Vector Control Division, Ministry of Health, Kampala, Uganda; 5 Department of Biological Science, Faculty of Science and Humanities, Shaqra University, Ad-Dawadimi, Saudi Arabia

**Keywords:** *Plasmodium*, *Schistosoma mansoni*, Real-time PCR, Surveillance, RDT, Faecal sampling

## Abstract

**Background:**

As part of ongoing co-surveillance of intestinal schistosomiasis and malaria in Ugandan school children, a non-invasive detection method for amplification of *Plasmodium* DNA using real-time (rt)PCR analysis of ethanol preserved faeces (EPF) was assessed. For diagnostic tabulations, results were compared to rtPCR analysis of dried blood spots (DBS) and field-based point-of-care (POC) rapid diagnostic tests (RDTs).

**Methods:**

A total of 247 school children from 5 primary schools along the shoreline of Lake Albert were examined with matched EPF and DBS obtained. Mean prevalence and prevalence by school was calculated by detection of *Plasmodium* DNA by rtPCR using a 18S rDNA Taqman^®^ probe. Diagnostic sensitivity, specificity, positive and negative predictive values were tabulated and compared against RDTs.

**Results:**

By rtPCR of EPF and DBS, 158 (63.9%; 95% CI 57.8–69.7) and 198 (80.1%, 95% CI 74.7–84.6) children were positive for *Plasmodium* spp. By RDT, 138 (55.8%; 95% CI 49.6–61.9) and 45 (18.2%; 95% CI 13.9–23.5) children were positive for *Plasmodium falciparum*, and with non-*P. falciparum* co-infections, respectively. Using RDT results as a convenient field-based reference, the sensitivity of rtPCR of EPF and DBS was 73.1% (95% CI 65.2–79.8) and 94.2% (95% CI 88.9–97.0) while specificity was 47.7% (95% CI 38.5–57.0) and 37.6% (95% CI 29.0–46.9), respectively. With one exception, school prevalence estimated by analysis of EPF was higher than that by RDT. Positive and negative predictive values were compared and discussed.

**Conclusions:**

In this high transmission setting, EPF sampling with rtPCR analysis has satisfactory diagnostic performance in estimation of mean prevalence and prevalence by school upon direct comparison with POC-RDTs. Although analysis of EPF was judged inferior to that of DBS, it permits an alternative non-invasive sampling regime that could be implemented alongside general monitoring and surveillance for other faecal parasites. EPF analysis may also have future value in passive surveillance of low transmission settings.

## Background

Malaria continues to be a public health problem in 90 countries worldwide [1], with the impact of global control flat-lining over the past 2 years; the greatest disease burden remains in sub-Saharan Africa (SSA) where over 90% of deaths occur [2]. Here, weak health systems with restricted diagnostic repertoires and inadequate access to prompt treatment preside, alongside favorable conditions for *Plasmodium* transmission [2, 3]. Collectively, this places certain demographical groups, e.g. infants, more vulnerable than others [4] although infection prevalence and non-severe disease in other groups, such school children, should not be overlooked [4–6]. A broader appraisal of infection surveillance is needed, especially in the future context of gaining transmission control and unveiling the importance of asymptomatic carriage [7–9]. Indeed, with introduction of more sensitive diagnostics, over and above what current rapid diagnostic tests (RDTs) can provide, our understanding of the environmental importance of asymptomatics will change [10]. Exploration of non-invasive sampling, e.g. collection of saliva or urine, for detection of *Plasmodium* DNA has been explored but as of yet is not routinely used [7, 11, 12].

In Uganda, for example, control of malaria is a major public health challenge [13], especially in young children [6]. The geographical distribution of malaria also aligns with other mosquito-borne diseases such as lymphatic filariasis, making co-surveillance with neglected tropical diseases justifiable [14]. On the Lake Albert shoreline, however, intestinal schistosomiasis is hyperendemic [15] and ongoing school-based preventive chemotherapy control has taken place, with periodic disease surveillance, over the last 15 years [16, 17]. The local prevalence of *Plasmodium falciparum* in mothers and pre-school-aged children is very high [18–20], with non-*P. falciparum* infections also reported [21] but the prevalence of infection in school children rarely measured [22]. As part of ongoing surveillance of intestinal schistosomiasis in this area, Al-Shehri et al. [15] revealed that over half of the sampled school children harboured RDT positive asymptomatic infections, a quarter being anaemic (< 115 g/L) and 11% had faecal occult blood in stool. The local prevalence of egg-patent intestinal schistosomiasis was 46.5%, ranging in excess of 80% in certain schools along the immediate shoreline [15].

Point-of-care (POC) and reference laboratory diagnostics tests play a critical role in monitoring and surveillance of disease control programmes, yet finding synergies for integrated surveillance is limited [23]. Indeed, current siloing of diseases and infection detection is unfortunate for there could be opportunities, where appropriate, to share programmatic costs and intervention repertoires for intestinal schistosomiasis and malaria [13, 24]. Faecal samples are widely collected for surveillance of intestinal schistosomiasis but have not been fully exploited to co-detect malaria, for example, by real-time PCR (rtPCR) analysis for *Plasmodium* DNA [25–27]. By contrast, non-invasive faecal sampling has burgeoned the detection of *Plasmodium* DNA in non-human primates, developing our epidemiological understanding of natural and zoonotic malaria immensely [28–31].

This study sought to investigate whether rtPCR analysis of extracted DNA from ethanol preserved faeces (EPF), collected during routine surveillance for intestinal schistosomiasis, could be informative for population surveillance of *Plasmodium* infection in school children.

## Methods

### Study area

In this cross-sectional survey, field sampling and examinations of children took place during May 2015 in five primary schools in Buliisa District located within the Lake Albert region. Three of these five schools have been visited previously as sentinel surveillance sites of the national control programme [32] and the global positioning system (GPS) coordinates for each school were recorded: Walakuba (GPS 01°50.323N, 031°22.740E), Bugoigo (GPS 01°54.004N, 031°24.750E) and Runga (GPS 01°43.828N, 031°18.603E) were located on the immediate shoreline at an approximate altitude of 615 m on average above sea level, while Biiso (GPS 01° 45.516N, 031°25.236E) and Busingiro (GPS 01°44.090N, 031°26.855E) were located over 10 km away inland with an altitude of 1295 m on average above sea level. This purposeful selection aimed to represent the current control landscape of preventive chemotherapy for schistosomiasis across high- and low-endemic settings and capture an altitudinal range where malaria transmission was suspected to vary.

### Sample collection and POC diagnosis of malaria

After obtaining written informed consent and verbal assent, a pre-target enrolment of 60 children per school, of equal gender, randomly recruited from classes primary 1 to 3, were assessed for study eligibility and requested to provide two stool samples on consecutive days, a single urine sample and a finger-prick blood sample. Point-of-care diagnosis of malaria was made according to an RDT (SD Bioline Malaria Ag P.f/Pan test, SD Diagnostics, Korea). A single drop of blood was spotted onto filter paper (Whatman 3 MM, Whatman International, Maidstone, England), dried at ambient temperature and sealed in a plastic bag with dessicant silica gel to preserve each DBS before transportation to the LSTM. DBS were then stored at − 20 °C before DNA extraction. Stool samples were filtered through a 212 µm metal mesh before 0.5 g were placed in 1 mL of 95% ethanol for the EPF before transfer to the LSTM and subsequent DNA extraction.

### Detection of *Plasmodium* DNA by rtPCR TaqMan™ assay

DNA extraction from EPF was conducted according to protocols described by Al-Shehri et al. following standard methods [33]. For DBS, DNA was extracted using Chelex Resin (Chelex^®^ 100 sodium form 50–100 mesh dry, No 11139-85-8, Sigma) [34]. Extracted DNA was quantified and then analysed using a TaqMan™ genus-specific probe targeting the ribosomal 18S DNA gene [35]. In a total of 25 μL in each reaction, consisting of 12.5 μL IQ supermix, 5 μL of DNA template, specific forward primer, Plasmo-2 R primer, generic Plasmo-probe, and the reaction volume was completed with nuclease-free H_2_0 (Sterile, RNase and DNase Free, 3098-2ML, UK). Extracted DNA-DBS and DNA-EPF for each school were run on different plates, included a number of controls: extraction of TE (10 mM Tris–HCl [pH 8], 1 mM EDTA) as a negative control, and four positive controls, using DNA extracted from reference *P. falciparum, Plasmodium vivax*, *Plasmodium ovale* or *Plasmodium malariae* infections as provided by the clinical diagnostic laboratory at LSTM. A 10% selection of experimental results were repeated to ensure assay reproducibility. DNA-TaqMan™ assays were performed in a Chromo-4 with Opticon monitor Version 3.1. (Biorad, Hemel Hempstead, UK) and thermal cycling conditions of an initial denaturation of 3 min at 95 °C, followed by 50 cycles of 15 s at 95 °C, 20 s at 60 °C and 15 s at 72 °C. Infected children were identified according to C_*t*_ values: either positive (10 > C_*t*_ ≤ 45) or negative (C_*t*_ > 45).

### Antiparasitic treatments offered

Each child was examined by the project nurse and offered praziquantel (40 mg/Kg, Merck KGaA, Darmstadt, Germany) and albendazole (400 mg, GSK, Brentford, UK) irrespective of their infection status, although children observed with a positive malaria RDT were each offered a take-home, 3-day course of artemether–lumefantrine (Coartem^®^ Lonart; Cipla, Mumbai, India) treatment with the first and third treatment administrations overseen by the project nurse. The Ugandan Council for Science and Technology and the Liverpool School of Tropical Medicine granted approvals for this study.

### Statistical analysis

Statistical and regression analysis was performed using Minitab Ltd^®^ (Brandon Court, Unit E1-E2 Coventry CV3 2TE UK), calculating prevalence, sensitivity, specificity, positive predictive value (PPV) and negative predictive value (NPV) of each of the diagnostic methods.

## Results

A total of 271 children were initially screened; 247/271 (91.1%) met the inclusion criteria of sample provision and obtaining verbal assent, were enrolled with mean age of 6.8 years (95% CI 6.6–6.9), with males accounting for 124/247 (50.2%), and females 123/247 (49.7%). Of 247 school-aged children that tested for malaria infection by RDTs using malaria Ag P.f/Pan test, 138 (55.8%; 95% CI 49.6–61.9) were positive for *P. falciparum* (*Pf*HRP-2-detecting RDTs), and 45 samples (18.2%; 95% CI 13.9–23.5) were positive for *Plasmodium* species (*Pf*HRP-II and pan-pLDH test lines). A singleplex rtPCR-based assay for DBS revealed that 198 (80.1%, 95% CI 74.7–84.6) children were positive for *Plasmodium* DNA whereas a singleplex rtPCR-based assay for EPF revealed that 158 (63.9%; 95% CI 57.8–69.7) children were positive, (Table 1).

**Table 1:** Prevalence and performance of diagnostic tests for detection of *Plasmodium* infection in school-aged children

School name	Walukuba (n = 43)	Runga (n = 50)	Bugoigo (n = 56)	Biiso (n = 49)	Busingiro (n = 49)	Total (n = 247)
	n (%)	n (%)	n (%)	n (%)	n (%)	n (%)
Prevalence						
Rapid diagnostic test^a^						
Negative	29 (67.4)	21 (42.0)	26 (46.4)	16 (32.6)	17 (34.6)	109 (44.1)
Positive	14 (32.5)	29 (58.0)	30 (53.5)	33 (67.3)	32 (65.3)	138 (55.8)
95% CI	[20.4–47.4]	[44.2–70.6]	[40.7–65.9]	[53.3–78.7]	[51.3–77.0]	[49.6–61.9]
rtPCR-based assays for DBS^b^						
Negative	10 (23.2)	8 (16.0)	24 (42.8)	5 (10.2)	2 (4.0)	49 (19.8)
Positive	33 (76.7)	42 (84.0)	32 (57.1)	44 (89.8)	47 (95.9)	198 (80.1)
95% CI	[62.2–86.8]	[71.4–91.6]	[44.1–69.2]	[78.2–95.5]	[86.2–98.8]	[74.7–84.6]
rtPCR-based assays for EPF^c^						
Negative	28 (65.1)	31 (62.0)	13 (23.2)	4 (8.1)	13 (26.5)	89 (36.0)
Positive	15 (34.8)	19 (38.0)	43 (76.7)	45 (91.8)	36 (73.4)	158 (63.9)
95% CI	[22.4–49.8]	[25.8–51.8]	[64.2–85.9]	[80.8–96.7]	[59.7–83.7]	[57.8–69.7]

No. of infected	(n = 33)	(n = 42)	(n = 32)	(n = 44)	(n = 47)	Total (n = 198)
Co-infection (SCH, MAL^d^)						
Negative	4 (12.1)	6 (14.2)	21 (65.6)	36 (81.8)	43 (91.4)	110 (55.5)
Positive	29 (87.8)	36 (85.7)	11 (34.3)	8 (18.1)	4 (8.5)	88 (44.5)
95% CI	[72.6–95.1]	[72.1–93.2]	[20.4–51.6]	[9.5–31.9]	[3.3–19.9]	[37.6–51.4]

^a^Malaria by RDT (SD Bioline Malaria Ag P.f/Pan test, SD Diagnostics, Yongin-si, Gyeonggi-do, Korea)

^b^A TaqMan-based real-time PCR assay that collectively detected all 4 *Plasmodium* species from DBS

^c^A TaqMan-based real-time PCR assay that collectively detected all 4 *Plasmodium* species from EPF

^d^Co-infections among schoolchildren with *Plasmodium *species detected by rtPCR-based assay for DBS and *Schistosoma mansoni* by Kato-Katz faecal microscopy

**Fig. 1:**
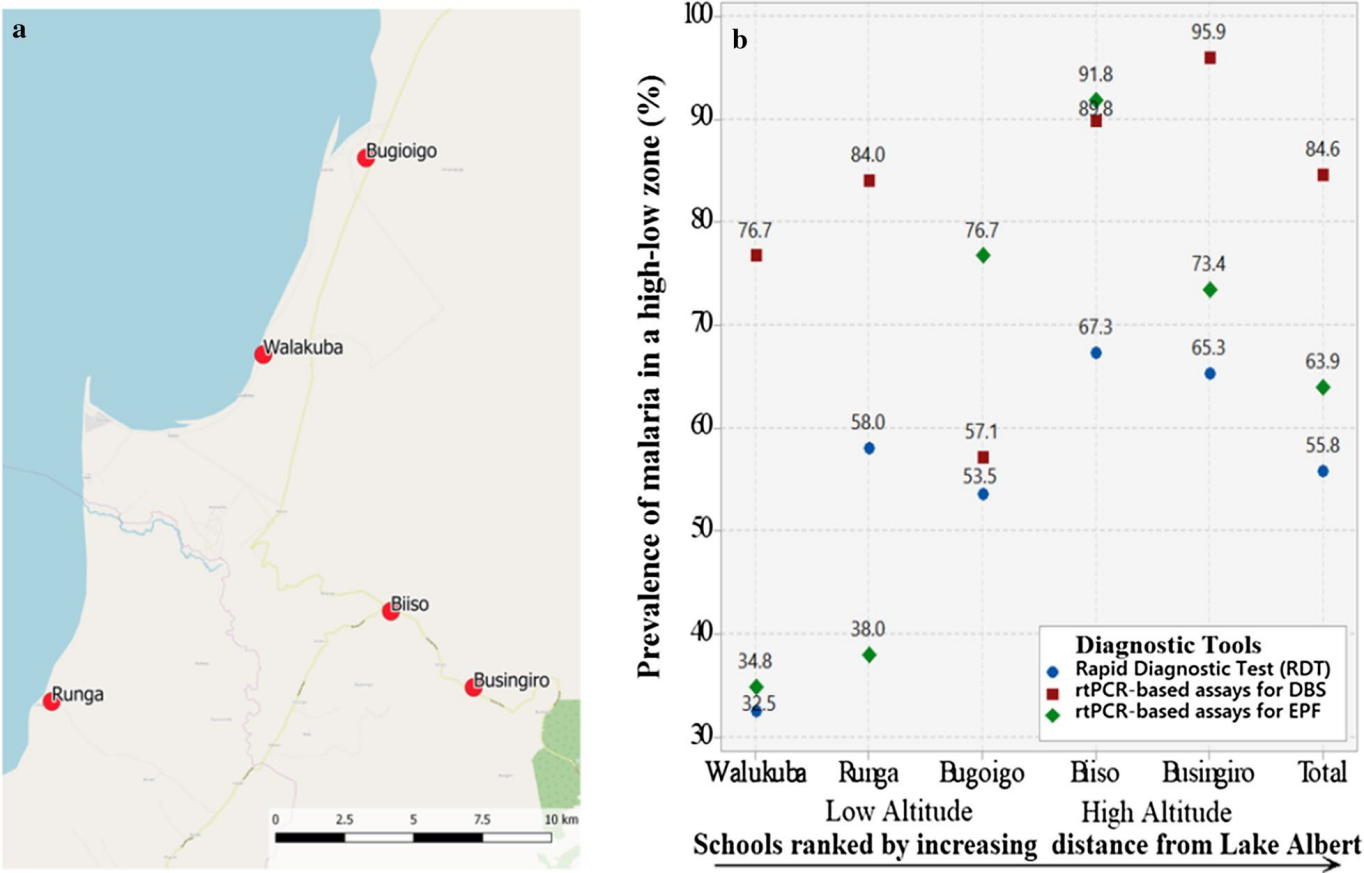
Location of 5 sampled schools and estimated prevalence of malaria. **a** Schematic map of the five sampled primary schools in the Lake Albert region, the blue area indicates Lake Albert. The primary schools of Bugoigo, Walakuba and Runga on the lakeshore plain (Low-Altitude), whereas Biiso and Busingiro are located on top of the escarpment (High-Altitude). Once part of Masindi District, the schools are now located within Buliisa District after administrative areas were revised. **b** Estimated prevalence of malaria infection by altitude and school distance from the shoreline for each examined diagnostic test

Although absolute values differed, the trends of *Plasmodium* prevalence across the three diagnostic methods by “Low–High Altitude” and “distance from Lake Albert” were similar. A positive result by any test increased significantly relative to distance from the lake (p < 0.001 for all three diagnostic tools) (Fig. 1). The highest prevalence of malaria infection was by DBS analysis of 95.9% (95% CI 86.2–98.8) at Busingiro and, for EPF, 91.8% (95% CI 80.8–96.7) at Biiso. At low altitudes and the shoreline schools of Walakuba and Runga, prevalence by RDT was 32.5% (95% CI 20.4–47.4) and 58.0% (95% CI 44.2–70.6), by rtPCR for EPF was 34.8% (95% CI 22.4–49.8) and 38.0% (95% CI 25.8–51.8), and for DBS was 76.7% (95% CI 62.2–86.8) and 84.0% (95% CI 71.4–91.6). In Bugoigo school, the prevalence of malaria by rtPCR from EPF appeared raised, 76.7% (95% CI 64.2–85.9), upon comparison to the other two diagnostic tools.

**Fig. 2:**
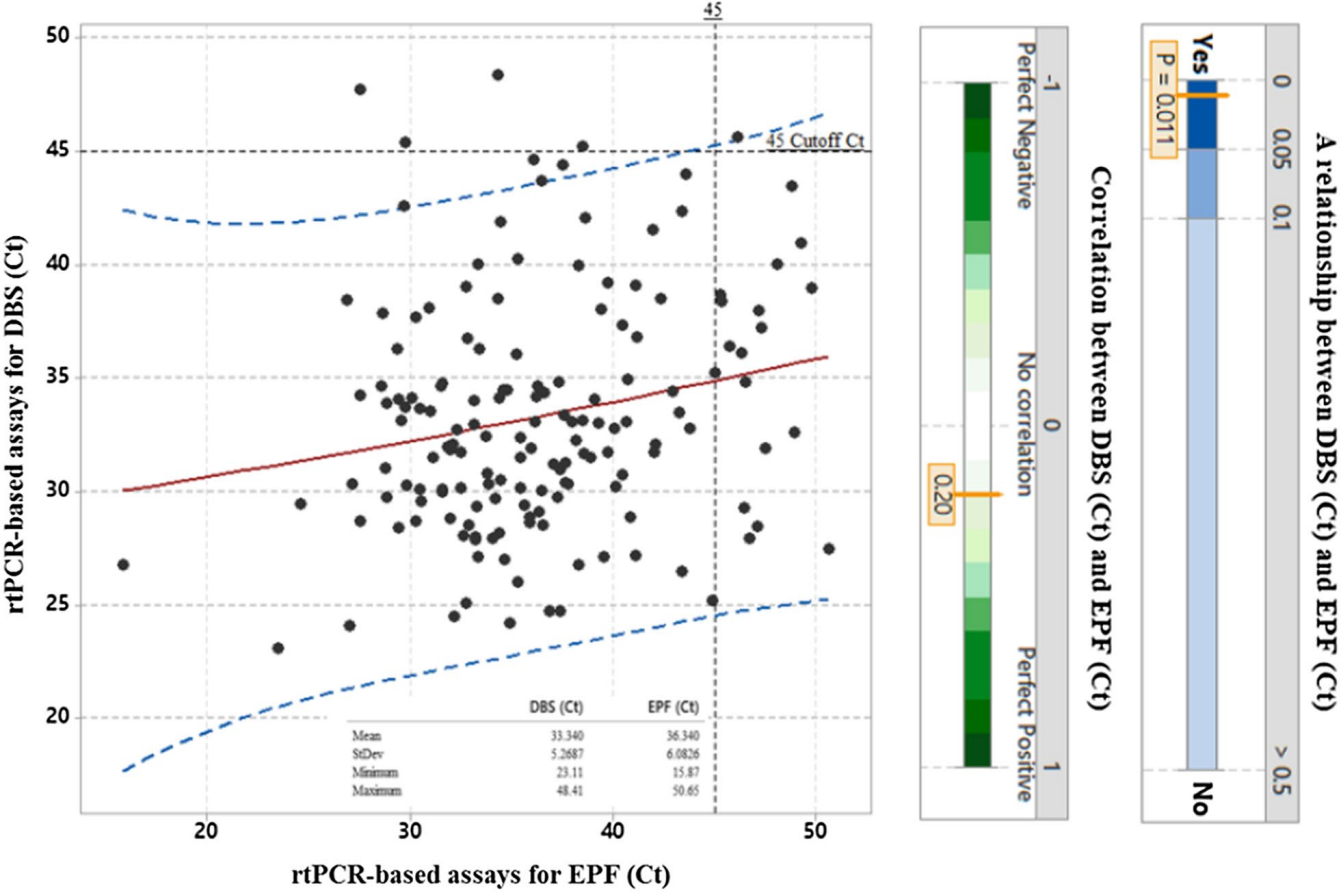
Showing the regression between rtPCR-based assays for DBS (C_*t*_) and rtPCR-based assays for EPF (C_*t*_) for the singleplex assay to detect generic *Plasmodium* (18S rDNA gene). The red fitted line shows the predicted value and cross line is the cut off 45 C_*t*_. The blue dashed lines show the 95% prediction interval. The mean C_*t*_ value of EPF (C_*t*_) is 36.3, and DBS (C_*t*_) is 33.3, and the actual difference between DBS (C_*t*_) and EPD (C_*t*_) is 2.3 (95% Cl 1.1–3.5)

The relationship between C_*t*_ value of rtPCR-based assays for DBS and C_*t*_ value for EPF was DBS (C_*t*_) = 26.93 + 0.1763 EPF (C_*t*_) and was statistically significant (*p* = 0.01), with R-squared 4.14% (*p* < 0.005). The positive correlation as shown r = 0.2 (Fig. 2), indicating that when EPF (C_*t*_) increased, so did the DBS (C_*t*_) (*p* < 0.05), although the magnitude of this change was not substantive. Using RDT results as convenient field-based comparison, the diagnostic score tabulations are presented (Table 2).

**Table 2:** Empirical estimates of sensitivity (SS), specificity (SP), negative predictive value (NPV) and positive predictive value (PPV), Cohen’s kappa for each diagnostic test against Malaria by RDT as ‘gold standard’

Assay	Negative (%)	Positive (%)	Total (%)	Estimate % (95% CIs)		Diagnostic accuracy (95% CIs)	Diagnostic odds (95% CIs)	Cohen’s kappa
Rapid diagnostic tests (RDTs)								
rtPCR of DBS^a^								
Negative	41 (83.6)	8 (16.3)	49 (19.8)	Sensitivity	94.2% [88.9–97.0]	69.2% [63.2–74.6]	9.7 [4.3–22.0]	0.3 [0.2–0.4]
Positive	68 (34.3)	130 (65.6)	198 (80.1)	Specificity	37.6% [29.0–46.9]			
Total (%)	109 (44.1)	138 (55.8)	247 (100.0)	PPV	65.6% [58.8–71.9]			
				NPV	83.6% [70.9–91.4]			
rtPCR of EPF^a^								
Negative	52 (58.4)	37 (41.5)	89 (36.0)	Sensitivity	73.1% [65.2–79.8]	61.9% [55.7–67.7]	2.4 [1.4–4.2]	0.2 [0.1–0.3]
Positive	57 (36.0)	101 (63.9)	158 (63.9)	Specificity	47.7% [38.5–57.0]			
Total (%)	109 (44.1)	138 (55.8)	247 (100.0)	PPV	63.9% [56.1–71.0]			
				NPV	58.4% [48.0–68.1]			

^a^rtPCR-based assays based on detecting a region of the *Plasmodium* 18S DNA gene that is conserved across all five species *P. falciparum*, *P. vivax*, *P. ovale* and *P. malariae*

## Discussion

In the present study, amplification of *Plasmodium* DNA from non-invasive EPF and invasive DBS sampling has shown that 158 (63.9%; 95% CI 57.8–69.7) and 198 (80.1%; 95% CI 74.7–84.6) of 247 children tested positive for infection (Table 1). As might be expected given the greater sensitivity of rtPCR methods based in the laboratory [7, 36, 37], the prevalence estimated by either EPF or DBS was higher than that by RDTs where 138 children (55.8%; 95% CI 49.6–61.9) were judged infected. Using the RDT, inspecting lactate dehydrogenase (pLDH) reactions, 45 infected cases (18.2%; 95% CI 13.9–23.5) of pan-*Plasmodium* spp. co-infections were found. The general agreement of infection mean prevalence and prevalence by school for EPF and RDT was satisfactory and would infer that this non-invasive sampling protocol yields very similar school-level information. A further diagnostic evaluation might consider inclusion of point-of-contract microscopy [38, 39], particularly when parasite densities are below RDT detection thresholds [37].

Taking altitude and distance from Lake Albert into account, the highest prevalence of *Plasmodium* infection by any method was at Busingiro school, where 47 out of 49 pupils (95.9%; 95% CI 86.2–98.8) tested positive by DBS sampling (Table 1). In contrast, 73.4% (95% CI 65.2–79.8) of the same pupils tested positive by EPF sampling while prevalence by RDT was 65.3% (95% CI 51.3–77.0). The highest prevalence by EPF sampling was at Biiso school, and in all but one school (Runga), both DBS and EPF sampling provided higher prevalence of infection that RDTs (Fig. 1). These findings confirm those by Jirků et al. [25] in that *Plasmodium* DNA shed at detectable levels by rtPCR can be readily found within faecal material.

Unlike in previous studies where *P. falciparum* infection and parasite density was negatively associated with rising altitude [40–43], this study found that the highest percentages of *Plasmodium* DNA were in higher altitude schools (Biiso and Busingiro) (Fig. 2b); in both schools, EPF sampling detected a higher percentage of infection than RDTs. At lower altitudes, the performance of EPF sampling was less predictable against the two other methods. It is worthy to note that egg-patent intestinal schistosomiasis is very common in these three schools [44] and may perhaps confound the detection of *Plasmodium* DNA. When determining the sensitivity and specificity of rtPCR-based assays; an initial evaluation of cycle threshold (C_*t*_) values showed that there was a positive correlation, although not particularly impressive, between DBS- and EPF-derived samples (r = 0.2), with a mean EPF Ct value of 36.3, and a mean DBS Ct value of 33.3, (Fig. 2). When correlating these results to RDTs, rtPCR-based assay of DBS samples showed a greater sensitivity than EPF samples (94.2% versus 73.1%), with rtPCR of DBS samples having a specificity of 37.6% and a PPV of 65.6%. In contrast, the specificity of EPF sampling was 47.7%, with a PPV of 63.9% (Table 2).

It is known that schistosomes immuno-modulate host responses which interplay with *Plasmodium* parasitaemia, often reducing numbers of *Plasmodium* within the blood [45]. This may lower the amount of *Plasmodium* DNA ultimately shed into the bowel but as schistosome eggs also directly perforate the bowel, and some 11% of children also had faecal occult blood in stool, thus there may be a more complicated relationship with detected *Plasmodium* DNA in faecal material than first assumed [25]. It remains to be assessed if this phenomenon also occurs in EPF sampling of non-human primates as intestinal schistosomiasis can naturally occur in these animals [46]. Using rtPCR with Taqman^®^ probes for *Schistosoma* boosts the diagnostic repertoire for detection of human and zoonotic schistosomiasis [44, 47] and could be also applied in non-human primate sampling.

Although faecal sampling has been a routine activity of the national control programme for bilharzia and intestinal worms for over a decade [48, 49], an immediate drawback in the use of EPF sampling is the need for a laboratory setting. Future advances in handheld rtPCR equipment may provide an alternative avenue by which EPF sampling becomes more portable and affordable in field conditions [7, 50, 51]. What is evident from the results of this study is that EPF sampling of *Plasmodium* infections in school children in this high transmission setting provides information without invasive sampling. Similarly, it has been shown that EPF sampling can shed light on the occurrence of other parasites and pathogens whose nucleic acids may be shed in stool [23]. Such passive collection of stools, or targetted collection for faecal parasites, could aid in developing a general monitoring and surveillance platform. For example, EPF sampling may have future application in evaluating dynamics of malaria in schools where intermittent preventive treatment has been allocated [51]. However, in Ebola-risk areas, due care should be taken in stool collection and processing [52]. With regard to malaria control in Uganda, EPF analysis could contribute to general surveillance of infection in schools [13], encouraging further integrated mapping initiatives with neglected tropical diseases [53], and assessing the future impact of intermittent preventive treatment initiatives [51].

## Conclusion

Examination of EPF by rtPCR analysis in this high transmission setting offers an attractive non-invasive sampling strategy for detection of *Plasmodium* DNA in school children; prevalence of infection by school was in close agreement with that obtained from POC-RDTs using finger prick blood. In future, using EPF and rtPCR analysis could be a powerful disease surveillance platform, able to synergize with helminth control programmes and likely prove useful in passive surveillance of infections within low transmission settings.

## Abbreviations

CI: confidence interval
DBS: dried blood spot
EPF: ethanol preserved faeces
RDT: rapid diagnostic tests
rtPCR: real-time polymerase chain reaction
